# TGF-β induces corneal endothelial senescence via increase of mitochondrial reactive oxygen species in chronic corneal allograft failure

**DOI:** 10.18632/aging.101659

**Published:** 2018-11-28

**Authors:** Zhiyuan Li, Ting Liu, Junwei Ma, Qie Guo, Liang Ma, Qiulan Lv, Yan Jiang, Chao Wei, Jisheng Zhang

**Affiliations:** 1 Key Laboratory, Department of Otolaryngology-Head and Neck Surgery, Affiliated Hospital of Qingdao University, Qingdao, China; 2 State Key Laboratory Cultivation Base, Shandong Provincial Key Laboratory of Ophthalmology, Shandong Eye Institute, Shandong Academy of Medical Sciences, Qingdao, China; 3 Department of Clinical Pharmacy, Affiliated Hospital of Qingdao University, Qingdao, China; 4 Department of Child Health Care, Affiliated Hospital of Qingdao University, Qingdao, China

**Keywords:** corneal endothelium, cellular senescence, transforming growth factor-β, mitochondrial reactive oxygen species, chronic corneal graft failure

## Abstract

The corneal endothelium (CE) dysfunction impairs optical transparency and leads to corneal allograft failure. Morphologically, CE cells are characterized by premature senescence at the late stage of corneal graft. However, the detailed molecular mechanisms are largely unknown. Here we found that transforming growth factor-β (TGF-β) is elevated in the CE of late graft failure. In addition, senescence-associated gene p21 and p16 are increased as well, which is consistent with their elevation upon TGF-β treatment in human corneal endothelial cell B4G12. Furthermore, TGF-β treatment leads to high positive ratio of SA-β-gal, indicating B4G12 cells undergo cellular senescence. Mechanistically, we demonstrated that TGF-β could induce mitochondrial ROS (mtROS) production and mtROS scavenger could rescue CE cell senescence upon TGF-β treatment. Our study provides new evidence that elevated TGF-β plays a crucial role in the CE cell senescence and loss in chronic corneal graft failure, which could be potential targets for clinical treatment.

## Introduction

The acute corneal allograft rejection has been improved signiﬁcantly over the last two decades, with an overall survival rate could reach 90% at first year. However, it drops to 74% in 5 years and only half sustain success within 10 years [1,2]. Chronic corneal allograft failure has become the major problem in clinical penetrating keratoplasty (PKP).

The corneal endothelium (CE) which locates in the inner surface and only contains a single cell layer with limited regenerative potential, maintains stromal dehydration via an ion pump mechanism [3]. Functional CE cells are essential to keep corneal graft transparency and long-term survival, while CE cell loss can cause corneal opacity overtime after PKP, which is substantial at 5 years post keratoplasty [4]. However, it remains poorly understood how the accelerated postoperative loss of CE cells occurs in chronic graft failure. It has fueled the desire to investigate the mechanisms responsible for chronic corneal allograft failure and develop clinically relevant protocols to prevent it.

Cellular senescence would contribute to irreversible proliferative arrest and may further result in functional losses of organs and reduced or lost vitality in organisms [5,6]. Oxidative stress and premature senescence were observed in the CE of chronic allograft failure [7]. Transforming growth factor-β (TGF-β) treatment led to senescence in diverse cell lines including tumor cells [8–10]. However, whether TGF-β affects premature senescence of CE cells during chronic corneal allograft failure has still not been elucidated.

In this study, we investigated the role of TGF-β in the CE of chronic corneal allograft failure. For the first time we found that TGF-β was substantially increased with CE cell senescence in chronic graft failure. Moreover, TGF-β treatment led to human CE cell senescence and senescence-associated inflammation. Mechanistically we demonstrated that TGF-β could induce mitochondrial ROS (mtROS) production and mtROS scavenger could rescue CE cell senescence upon TGF-β treatment. Our findings provide new evidence that elevated TGF-β plays a crucial role in the CE cell senescence and loss in chronic graft failure, which sheds light on the challenges of drug development for the penetrating keratoplasty at the late stage.

## RESULTS

### Increased cellular senescence in the human CE with chronic corneal graft failure

**Figure 1:**
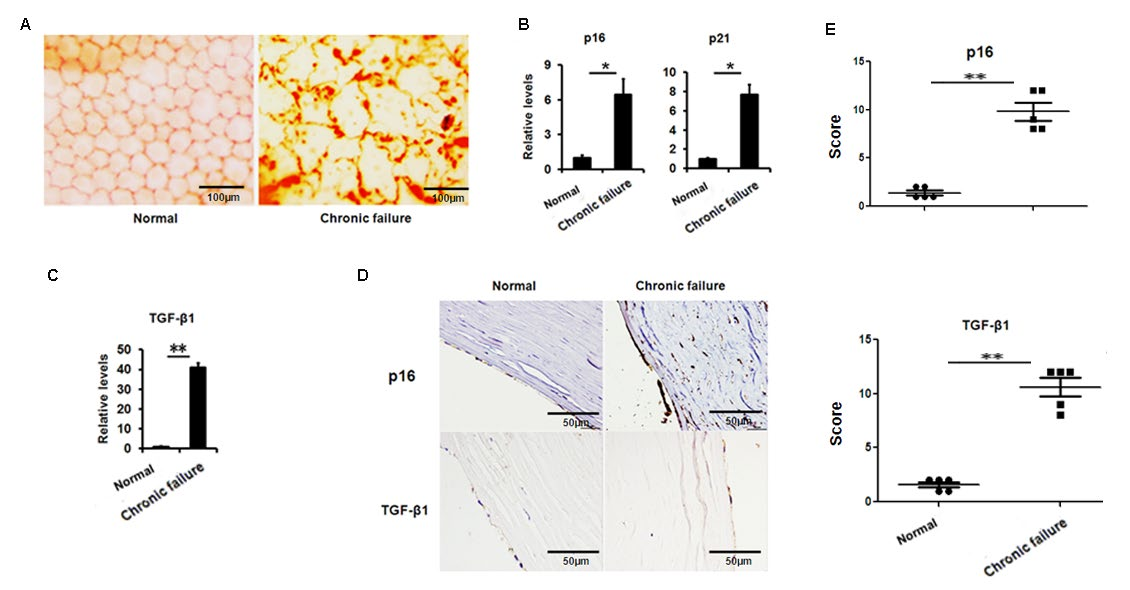
**Increased senescence associated markers and TGF-β1 in the human corneal buttons CE with chronic graft rejection.** (**A**) Clinical evaluation of corneal endothelial cells with chronic failure using alizarin red staining. (**B-C)** The mRNA expression of p16, p21 (**B**) and TGF-β1 (**C**) in the CE with or without chronic graft failure (**P<0.01,*P<0.05). (**D-E**) Representative photographs (**D**) and histopathology scores (**E**) for the IHC staining of p16 and TGF-β1 in the CE with chronic failure.

Alizarin Red Staining showed that the original hexagonal structure of endothelial cells with chronic graft failure was destroyed compared to that in healthy donor (Fig.1A). Accumulative evidence indicated that cellular senescence is closely related to organ transplant and involved in the pathogenesis of chronic graft failure [11,12]. Therefore, we speculate that cellular senescence was associated with the changes of corneal endothelial cells in chronic corneal graft failure. Our results showed that senescence-related genes p16 and p21 were dramatically increased at transcription level or protein level (Fig.1B, D, E), suggesting that decreased corneal endothelial cells may result from increased cellular senescence in the CE with corneal graft failure.

### Elevated expression of TGF-β1 in the human CE with chronic graft failure

Next, we questioned what triggered cellular senescence. A large body of evidence indicates that TGF-β signaling is involved in ocular diseases and cellular senescence. Therefore, we analyzed the expression of TGF-β in the CE. We found that TGF-β1 expression was largely elevated at the transcriptional level (Fig.1C) and protein level (Fig. 1D, E) in senescent CE compared to control. These results suggest that the increased TGF-β found in the CE with chronic graft failure may correlate to the increased cellular senescence.

### TGF-β1 induces senescence of B4G12 cells

**Figure 2:**
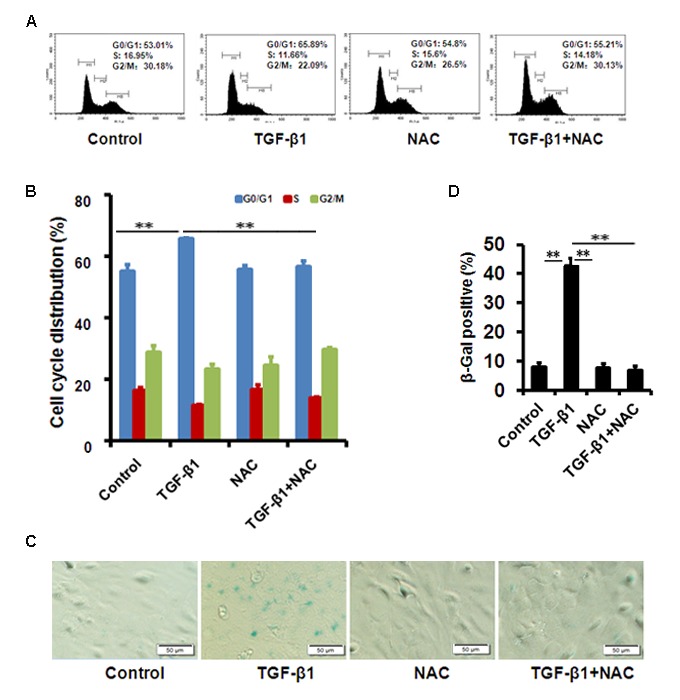
**TGF-β1 induces cellular senescence in B4G12 cells.** (**A-B**) The G1 phase arrest was induced by TGF-β1 treatment. Control and TGF-β1–treated B4G12 cells were subjected to cell cycle analysis after 48h-treatment. A representative ﬂow cytometric analysis of the DNA content was shown in (A) and the values are mean±SD in (**B**). (**C-D**) SA-β-Gal activity was measured in B4G12 cells treated with 10 ng/ml TGF-β1 alone, or in combination with NAC (10mM) for 72h. NAC treatment alone (10mM) did not affect the cell cycle status and SA-β-Gal activity. Bar graphs represent mean±SD. *P<0.05, **P<0.01. All the experiments were independently repeated at least three times.

**Figure 3:**
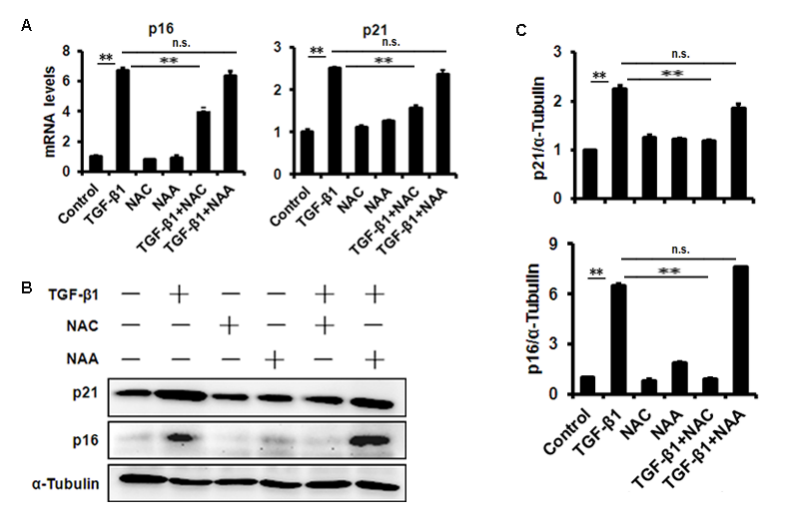
**TGF-β1 treatment of B4G12 cells causes the induction of p16 and p21.** B4G12 cells were treated with 10 ng/ml TGF-β1 alone, or in combination with NAC (10mM) or NAA (10mM) for 72h. The mRNA (**A**) and protein (**B-C**) expression of p16 and p21 were induced by TGF-β1. NAC or NAA treatment alone (10mM) did not change the levels of p16 and p21. Bar graphs represent mean±SD. *P<0.05, **P<0.01.

To evaluate the effect of TGF-β1 on corneal endothelium senescence, CE cell line B4G12 was used *in vitro*. Cellular senescence is characterized as an irreversible arrest of mitotic cells at G1 phase, while some cancer cells enter senescence at G2 or S phase. Our results showed that the B4G12 accumulated at G1 phase (from 53.01% to 65.89%) with a concomitant depletion of S phase cells (from 16.95% to 11.66%) upon TGF-β1 treatment (Fig. 2A, B), suggesting that cell cycle arrest during TGF-β1 induced CE cells senescence occurred at G1 phase. In addition, percentage of SA-β-gal–positive cells was upregulated upon TGF-β1 treatment (Fig. 2C, D). Moreover, TGF-β1 could increase the expression of p16 and p21 at mRNA and protein level (Fig. 3A, B, C). Taken together, our findings suggested that TGF-β1 was capable of inducing human CE cells senescence.

Senescent cells will develop a complex senescence-associated secretory phenotype (SASP). It has been demonstrated that increased production of inflammatory cytokines, such as interleukin (IL)-6 and -8, during senescence play substantial roles in the establishment and maintenance of senescent phenotype. To test whether SASP happens during TGF-β1-induced human CE cells senescence, we further measured the key inflammatory mediators. In TGF-β-induced senescent CE cells, elevated monocyte chemotactic protein-1 (MCP-1), IL-6 and tumor necrosis factor-α (TNF-α) gene expression (Fig. S1A) and secretion (Fig. S1B) was detected.

### TGF-β1 treatment enhances mtROS production in B4G12 cells

**Figure 4:**
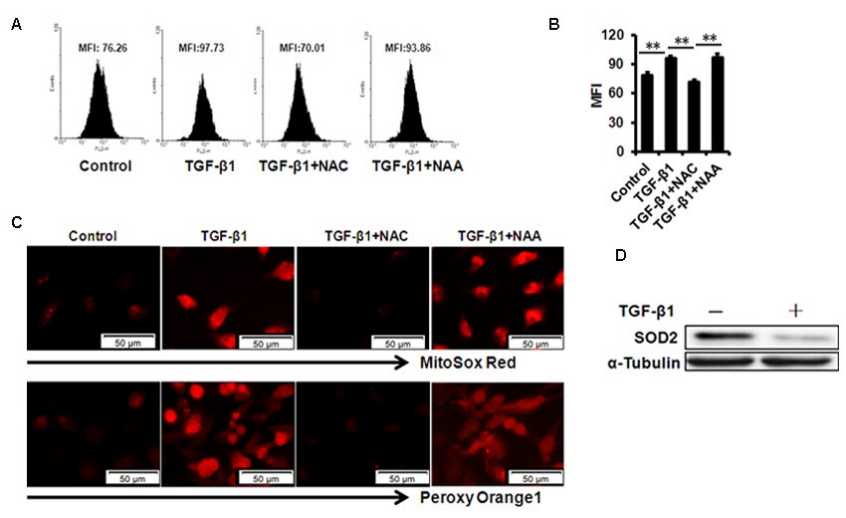
**mtROS production in B4G12 cells after exposure to 10ng/ml TGF- β 1 for 48 hours.** (**A-B**) Control and TGF-β1–treated B4G12 cells were subjected to MitoSOX Red Indicator staining after 48h of culture. A representative ﬂow cytometric analysis of mtROS was shown in (A) and the values are mean±SD (B). (**C**) The MitoSOX Red and peroxy orange 1 fluorescence was imaged with a fluorescent microscope. (**D**) SOD2 protein expression was tested in CE cells upon 10 ng/ml TGF-β1 treatment. *P<0.05, **P<0.01. All the experiments were independently repeated at least three times.

Mitochondrial ROS has been demonstrated to be involved in cellular senescence. Our results showed that mtROS were dramatically augmented in B4G12 after treatment of 10 ng/ml TGF-β1 for 48 hours detected with MitoSox Red by flow cytometer (Figure 4A and B). Similar results were obtained by fluorescent microscope (Fig. 4C). We also used the hydrogen peroxide- specific peroxy orange 1(PO1) probe to detect H_2_O_2_ molecule, and the use of this ROS probe confirmed the induction of ROS by TGF-β1(Fig. 4C). Next, we tested the expression of mitochondrial matrix enzyme Superoxide Dismutase2 (SOD2), which has been indicated as a mitochondria protector against oxidative radicals by suppressing mtROS production. Our results demonstrated that SOD2 expression was substantially decreased upon TGF-β1 treatment for 48 hours in B4G12 (Fig. 4D), which suggested its involvement in cellular senescence.

### NAC rescues TGF-β1 induced B4G12 cells senescence by repressing mtROS production

We further used N-acetyl-L-cysteine (NAC) as a physiological mtROS scavenger to rescue TGF-β1-induced senescence. Our data showed that 10 mM NAC could effectively inhibit mtROS production induced by TGF-β1 in B4G12 cells, while 10mM N-acetyl-L-alanine (NAA), a compound with similar chemical structure to NAC, was with no anti-ROS activity (Fig. 4A, B and C). In addition, NAC also reversed TGF-β1-induced G1 arrest (Fig. 2A, B), and markedly inhibited TGF-β1-induced SA-β-Gal activity (Fig. 2C, D), p16 and p21 expression (Fig. 3 A, B, C), as well as SASP production (Fig. S1A, B), whereas NAA failed to protect B4G12 cells from the senescence induced by TGF-β1 (Fig. 3 A, B, C). Taken together, our results suggest a key role for mtROS in TGF-β1-induced cellular senescence.

### Presence of immune cells on mouse CE with chronic graft failure

**Figure 5:**
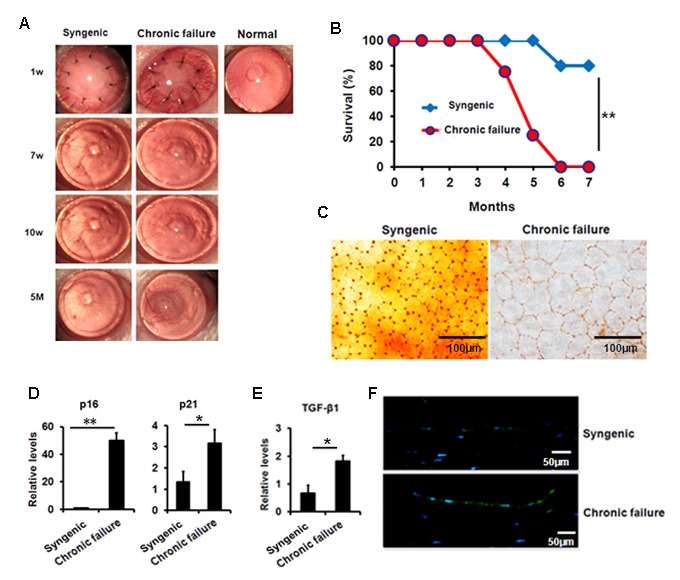
**Premature senescence and elevated TGF-β1 in the murine CE with chronic failure.** (**A-C**) Corneal graft phenotype in syngenic and chronic failure mouse model (**D-E**) p21, p16 (**D**) and TGF-β1 (**E**) expression was measured at transcription level in the murine CE from syngenic and chronic failure group. (**F**) Representative photographs for the IF staining of TGF-β1 in the CE with chronic graft failure. **P<0.01,*P<0.05.

**Figure 6:**
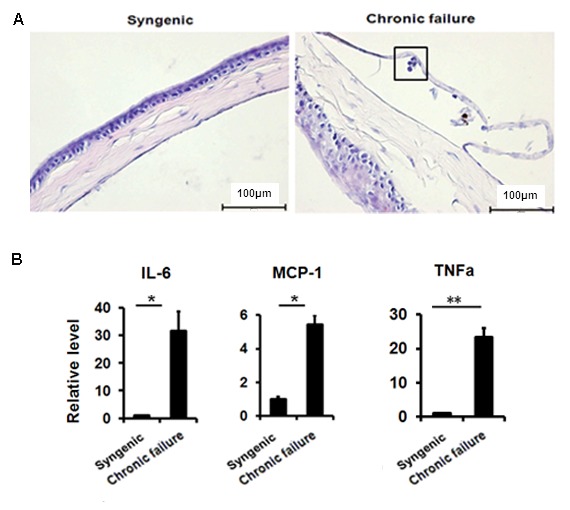
**Leukocytes in proximity to murine CE of chronic graft failure.** (**A**) H&E staining showing immune cell infiltration in the murine CE. (**B**)The expression of senescence-associated secretory phenotypefactor in RNA pooled from murine CE from chronic failure group compared with syngenic group. *P<0.05, **P<0.01. All the experiments were independently repeated at least three times.

Using a murine chronic allograft failure model in which graft failure occurs more than 90 days post-engraftment (Fig. 5A, B), we not only found the destroyed hexagonal structure of corneal endothelial cells from failed corneal grafts (Fig. 5C), but also found increased transcriptional levels of p16, p21 (Fig. 5D) and TGF-β1 (Fig. 5E) in the CE cells with graft failure, as well as shown in protein levels using immunofluorescent staining (Fig. 5F). In addition, in late failed corneal allograft group sporadic leukocytes were observed in proximity of CE (Fig. 6A), which were not found in the syngenic group. We speculated that CE cells may function as chemoattractant. Next, we measured pro-inﬂammatory factors associated with SASP by qPCR in CE cells from chronic failure and syngenic mouse. Elevated IL-6, MCP-1 and TNF-α gene expression (Fig. 6B) were observed in the chronic failure CE cells. Collectively, these results suggest that TGF-β could induce CE cell premature senescence and thus, contribute to CE cell accelerated loss in chronic corneal graft failure.

## DISCUSSION

Dysfunction of donor corneal endothelium becomes the predominant cause of graft failure after 5 postoperative years. As experimental and clinical evidence has shown, the structure and function of the CE cells are altered with premature senescence in chronic graft failure [13,14]. However, the underlying mechanisms of premature senescence in CE cells are largely unknown. Here, we showed that TGF-β1 expression is increased in CE cells of chronic failure. We also found that excessive TGF-β1 can lead to senescence of CE cells by inducing mtROS production.

Human endothelial cells function as "ion pump" to maintain the corneal transparency. Generally endothelial cells are arrested in G1 phase appearing as non-replicative state in vivo [15]. Recently increasing evidence has proved that corneal endothelial cells were repaired to keep monolayer integrity mainly though cell migration and enlargement [16,17]. Interestingly, *ex vivo* studies showed that comparing to younger donors endothelial cells from older donors have a longer G1-phase and requires stronger mitotic stimulation to complete the cell cycle, demonstrating senescent cells display aging-related dysfunction with gradual loss of proliferative ability. Of the mitotic stimulators TGF-β has shown to suppress cultured endothelial cell S-phase entry, implying TGF-β could repress proliferation and promote senescence *in vivo* [16–18]. Studies have demonstrated that TGF-β is able to result in senescence in a variety of normal tissue and tumor cells [9,10]. In this study we were focused on the role of TGF-β in chronic corneal graft failure. Our results demonstrated that TGF-β1 treatment greatly activate SA-β-Gal in CE cells, indicating CE cell senescence related phenotypes are controlled by TGF-β1. Premature senescence would lead to function changes of CE cells, which affects corneal graft transparency and long-term survival. Therefore, appropriate TGF-β suppression would benefit long term outcomes of corneal allograft.

It is known that mtROS is involved in cellular senescence. Previous study showed that intermediate concentrations of H_2_O_2_ could stimulate CE cells undergoing oxidative stress and cause senescence [7]. In our study, mtROS was notably induced by TGF-β1 in CE cells. Meantime, we found that SOD2, a key mitochondrial protecting enzyme against ROS [19], was drastically decreased in CE cells with treatment of TGF-β1. Importantly, mtROS scavenger NAC could rescue TGF-β1-induced senescence phenotype by inhibiting mtROS production. These data has strongly indicated TGF-β1 and its downstream mtROS could be potential therapeutic targets for chronic corneal allograft failure. This study shed a light on corneal transplant failure. However, it is still necessary to largely explore TGF-β1 network in regulating CE cell pathology after transplantation.

Senescence-associated secretory factors are usually released by senescent cells to reinforce senescence. In the present study, senescence-associated inflammation happens during TGF-β1-induced cellular senescence in human CE cells. Elevated MCP-1 and IL-6 gene expression and secretion was induced, as well as tumor necrosis factor alpha. In murine model for chronic allograft failure we also found increased cellular senescence and elevated TGF-β1 in murine CE cells of chronic failure. More interestingly, occasional leukocytes were presented in proximity to CE of late failure cornea, along with elevated levels of MCP-1, IL-6 and TNF-α. MCP-1 is a chemokine that regulates migration and infiltration of monocytes/macrophages [20,21], which may contribute to the infiltration of immune cells on chronic failure mouse CE cells, and reﬂect the response to accelerated CE cell loss in late failure mice. Our results have proved that SASP could be improved by excess TGF-β1 via increase of mtROS production in the CE of chronic failure. Our findings enrich the understanding of how the premature senescence of CE cells occurs in chronic corneal allograft failure, thereby providing potential therapeutic targets for chronic graft failure.

In summary, our study shows that excess TGF-β in CE cells of chronic graft failure can induce cellular senescence and SASP via increase of mtROS production. Our findings enrich the understanding of how the premature senescence of CE cells occurs in chronic corneal allograft failure, thereby providing potential therapeutic targets for chronic graft failure.

## MATERIALS AND METHODS

### Human cornea tissue

This study was approved by the Affiliated Hospital of Qingdao University Review Board and Shandong Eye Institute Review Board according to the tenets of the Declaration of Helsinki. Ten fresh chronic dysfunction corneal buttons more than 5 years after the first corneal transplantation were obtained prospectively. The sex-matched healthy corneas were obtained from International Federation of Eye Banks, Eye Bank of Shandong (Qingdao, China) and the donor corneal rims (residual tissues) were collected by penetrating keratoplasty (PKP) in this study.

### Animals

The animal experiment procedure was approved by Institute Animal Care and Use Committee of Qingdao University according to National Institutes of Health guide for the care and use of Laboratory animals. Male BALB/c and C57BL/6 (B6) mice were obtained from Shanghai Experimental Animal Center. All mice were cultivated under SPF condition. Mice used for corneal transplantation were between 8 and 12 wk of age.

### Corneal transplantation and evaluation of grafted mouse corneas

B6 corneas were grafted onto the right eye of BALB/c recipients for allogenic, as described previously [22]. In brief, Donor grafts were placed into the recipients and the sutures were taken out in one week after corneal transplant (Mani, Tochigi, Japan). Clinical examination was performed two times once a week. The grafts were scored for opacity as previously described [22]. At each time point, graft opacity needs to be evaluated on a scale of 0–5. Grafts were considered to be undergoing acute rejection with persistent opacity score > 2 within 2–5 weeks postoperatively, while grafts which were clear through 5–6 weeks postoperatively but developed opacity at > 90 d were considered as chronic graft failure.

### Cell culture and treatment

Immortalized CE cell line B4G12 was maintained in K-SFM (Gibco, Grand Island, NY) containing 2% FBS within 5% CO_2_ at 37°C. Medium was replaced by fresh serum-free K-SFM with 10 ng/ml recombinant human TGF-β1 (Peprotech) alone, or in combination with NAC (a potent ROS scavenger, 10 mM, Sigma) or NAA (10 mM, Sigma) after overnight starvation.

### Immunohistochemistry and immunoﬂuorescence assay

Immunohistochemical and immunoﬂuorescence assays were conducted as previously described [23]. In brief, paraffin embedded corneas was sectioned for 4 μm and antigen retrieval was possessed (Maixin Biotech, Fujian, China). TGF-β1 antibody (sc146, Santa Cruz) was used as primary antibody and naive IgG of appropriate specie was used as negative control.

### Cell Cycle analysis

Cell cycle status in the B4G12 cells was detected by measuring nuclear DNA content. The cells were collected 48 hours after exposure to TGF-β1 and were ﬁxed in 70% ethanol at 4°C for more than 2 hours. The pellet was collected by centrifugation before the RNase A solution was added (10 mg/ml). After a 1-hour incubation at 37°C, the cells were stained with propidium iodide (PI; ﬁnal concentration 100 μg/ml) at 37°C for 30 minutes. The samples were analyzed using a FACS Calibur ﬂow cytometer (BD Bioscience).

### Mitochondrial ROS measurement

Mitochondrial ROS (mtROS) were measured using a MitoSOX ™ Red mitochondrial superoxide indicator (Invitrogen), or hydrogen peroxide- specific peroxy orange 1(PO1, APExBIO) probe as the manufacturer’s instructions. Briefly, TGF-β1 treated B4G12 cells were incubated with 5 μM MitoSOX red for 10 min, or 5 μM PO1 for 30 min in the incubator. After removing the reagents and washing twice, fluorescence was detected with a FACS Calibur ﬂow cytometer (BD Bioscience) or a fluorescent microscope.

### Quantitative RT-PCR

Total RNA was extracted from the cornea endothelium or cultured B4G12 cells using TRIzol reagent (Invitrogen, Carlsbad, CA, USA). Then, cDNAs were obtained with PrimeScript RT reagent Kit (TOYOBO, Osaka, Japan). All the qPCR was done with SYBR GREEN reagents at ABI prism 7500 (Applied Biosystems, Foster City, CA, USA). Primers were shown in the Table1.

**Table1:** Primers used for real time-PCR.

**Gene name**	**Orientation**	**Primer sequence (5' - 3')**
GAPDH^1^	forward	AGGGCTGCTTTTAACTCTGGT
	reverse	CCCCACTTGATTTTGGAGGGA
P16^1^	forward	CACGGGTCGGGTGAGAGT
	reverse	CCCAACGCACCGAATAGTTAC
P21^1^	forward	GCCTGGACTGTTTTCTCTCG
	reverse	ATTCAGCATTGTGGGAGGAG
TGF-β1^1^	forward	CCCAGCATCTGCAAAGCTC
	reverse	GTCAATGTACAGCTGCCGCA
IL-6^1^	forward	GATGAGTACAAAAGTCCTGATCCA
	reverse	CTGCAGCCACTGGTTCTGT
MCP-1^1^	forward	CTCCAAGGGCCTCCTCTAC
	reverse	AAGGGCGAGATGACTCTGAA
TNF-α^1^	forward	CAGCCTCTTCTCCTTCCTGAT
	reverse	GCCAGAGGGCTGATTAGAGA
GAPDH^2^	forward	ACGGCAAATTCAACGGCACAGTCA
	reverse	TGGGGGCATCGGCAGAAGG
P16^2^	forward	GTGTGCATGACGTGCGGG
	reverse	GCAGTTCGAATCTGCACCGTAG
P21^2^	forward	TGCCAGCAGAATAAAAGGTG
	reverse	TTGCTCCTGTGCGGAAC
TGF-β1^2^	forward	CAACGCCATCTATGAGAAAACC
	reverse	AAGCCCTGTATTCCGTCTCC
IL-6^2^	forward	GATGGATGCTACCAAACTGGAT
	reverse	CCAGGTAGCTATGGTACTCCAGA
MCP-1^2^	forward	GCTCAGCCAGATGCAGTTAA
	reverse	TCTTGAGCTTGGTGACAAAAACT
TNF-α^2^	forward	AATGGCCTCCCTCTCATCAGT
	reverse	GCTACAGGCTTGTCACTCGAATT

^1^ human; ^2^ mouse

### Western blot

For whole cell extraction, the B4G12 cells were lysed with RIPA buffer (Beyotime, P0013B, Beijing, China). The detailed procedure has been previously described [9]. The primary antibodies were used as following: α-tubulin (11224-1, Proteintech), p16 (ab108349, Abcam), p21 (ab109520, Abcam) and SOD2 (ab13533, Abcam).

### SA-β-Gal staining and ELISA

SA-β-gal activity was detected with Staining Kit according to manufacturer’s instructions (Beyotime, RG0039, Beijing, China). Levels of IL-6 and MCP-1 in cell culture supernatants were measured using commercially available enzyme-linked immunosorbent assay (ELISA) kits (eBioscience).

### Statistical analyses

All experiments were performed at least three times independently, and data was presented as mean±SD. Two-tailed Student’s t-test and one-way analysis of variance (ANOVA) were used for two groups and more was used for statistical analyses. p<0.05 was considered statistically significant.

## SUPPLEMENTARY MATERIAL

Figure S1
