# Supplementary material for: TGF-β induces corneal endothelial senescence via increase of mitochondrial reactive oxygen species in chronic corneal allograft failure
Source: Aging (Albany NY). 2018 Nov 28;10(11):3474–85. doi: 10.18632/aging.101659 (PMC6286827; doi:10.18632/aging.101659)
Supplement: Figure S1 [file aging-10-101659-s001.pdf]

SUPPLEMENTARY MATERIAL

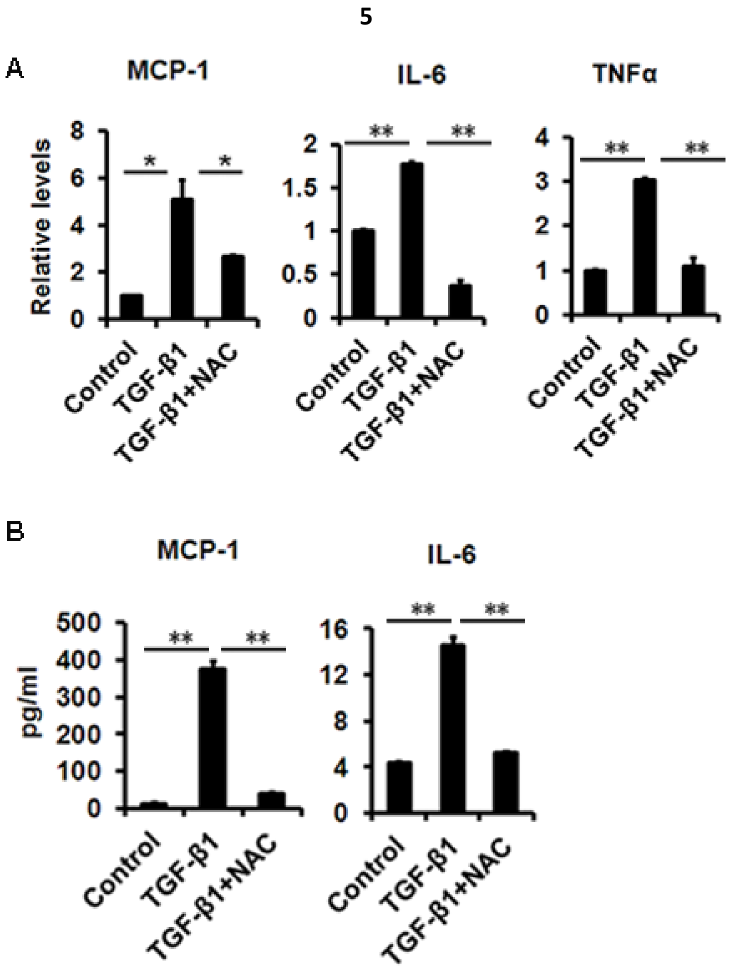

Figure S1. Effects of TGF-β1 on SASP production. (**A**) mRNA expression was measured by RT-qPCR in CE cells upon treatment of 10ng/ml TGF-β1 for 72h. (**B**) The MCP-1 and IL-6 in cultured CE cells supernatants were detected by ELISA. **P*<0.05, ***P*<0.01. All the experiments were independently repeated at least three times.
